# Respiratory rate modulation improves symptoms in patients with pulmonary hypertension

**DOI:** 10.1177/20503121211053930

**Published:** 2021-10-29

**Authors:** Barbro Kjellström, Bodil Ivarsson, Lise-Lotte Landenfelt Gestré, Henrik Ryftenius, Magnus Nisell

**Affiliations:** 1 Department of Clinical Sciences, Lund University and Clinical Physiology, Skåne University Hospital, Lund, Sweden; 2 Cardiology Unit, Department of Medicine, Karolinska Institutet, Stockholm, Sweden; 3 Department of Clinical Sciences, Lund University, Cardiothoracic Surgery and Medicine Services University Trust, Region Skåne, Lund, Sweden; 4 Lung Unit, Karolinska University Hospital and Department of Medicine, Karolinska Institutet, Stockholm, Sweden

**Keywords:** Rare diseases, chronic illness, pulmonary arterial hypertension, chronic thromboembolic pulmonary hypertension

## Abstract

**Background:**

Pulmonary arterial hypertension and chronic thromboembolic pulmonary hypertension are chronic diseases with a severe symptom burden. Common symptoms are dyspnoea at light activity and general fatigue that limits daily activities. Respiratory modulation by device-guided breathing decreased symptoms in patients with heart failure. The aim of this pilot study was to investigate if respiratory modulation could improve symptoms of dyspnoea in patients with pulmonary arterial hypertension or chronic thromboembolic pulmonary hypertension.

**Method:**

Adult patients with pulmonary arterial hypertension or chronic thromboembolic pulmonary hypertension with symptoms of dyspnoea at rest or light activity performed home-based respiratory modulation by device-guided breathing 20 min a day for 3 months. Patients were on stable disease-specific treatment ⩾3 months and willing to undergo all study procedures. Dyspnoea score, World Health Organization class, physical status, N-terminal pro b-type natriuretic peptide, quality of life, respiratory rate and 6-min walk distance were assessed before and after 3 months with respiratory modulation.

**Results:**

Nine patients with pulmonary arterial hypertension and five with chronic thromboembolic pulmonary hypertension completed the study protocol. Mean age was 71 ± 14 years, and 11 were women. After 3 months of respiratory modulation, dyspnoea score (−0.6, *p* = 0.014), respiratory rate at rest (−3 breaths/min, *p* = 0.013), World Health Organization class (−0.3, *p* = 0.040), quality of life (EuroQol Visual Analogue Scale +5 points, not significant) and decreased N-terminal pro b-type natriuretic peptide (−163 ng/L, *p* = 0.043) had improved. The fatigue and respiratory rate after the 6-min walk decreased while the 6-min walk distance remained unchanged.

**Conclusion:**

Patients with pulmonary arterial hypertension or chronic thromboembolic pulmonary hypertension that used device-guided breathing for 3 months improved symptoms of dyspnoea and lowered the respiratory rate at rest and after exercise.

## Introduction

Pulmonary arterial hypertension (PAH) and chronic thromboembolic pulmonary hypertension (CTEPH) are characterized by vasoconstriction and vascular proliferation of the small pulmonary arteries, leading to increased pulmonary vascular resistance and increased pulmonary artery pressures.^1^ The increased right ventricular (RV) afterload will induce RV dilatation and subsequent RV hypertrophy followed by RV failure and death. Drug treatments^1^ have improved both survival and quality of life (QoL) for these patients, but life expectancy still remains short and QoL impaired.^2,3^ Unfortunately, shortness of breath, dyspnoea on exertion and general fatigue are the symptoms that often remain despite haemodynamically successful treatment.^2^ Thus, patients with PAH or CTEPH might need additional treatment alternatives directed specifically at their main symptom, dyspnoea.

Dyspnoea might have several origins and in patients with right or left ventricular failure, a common reason is pulmonary oedema. In addition, the enlarged heart can in itself cause limitations in pulmonary function.^4^ These causes are often posture dependent and symptoms will worsen in a supine position.^5^ A sensitized chemoreflex response due to vasoconstriction and slower circulation through chemoreflex areas might also cause dyspnoea.^6^ In patients with PAH, a decreased strength in inspiratory muscles has also been described as a reason for dyspnoea.^7,8^

Respiratory rate modulation (RRM) by device-guided breathing (DGB) aims to consciously slow the respiratory rate. DGB has been shown to give a feeling of relaxation and well-being as well as decrease blood pressure in patients with hypertension^9^ and decrease symptoms of dyspnoea and fatigue in patients with heart failure and patients with PAH.^10,11^ The aim of the present pilot study was to investigate if RRM using DGB could decrease symptoms of dyspnoea in patients with PAH or CTEPH.

## Methods and material

### Study design

The study was a single-centre, prospective and single arm study conducted during 2015 and 2016. The study should be regarded as a pilot study. The intervention was a home-based RRM exercise performed 20 min, once a day and for 3 months. The study-related measurements were performed at start (baseline), after 3 months using the RRM (intervention) and after 3 months without the RRM (end).

The study was approved by the Regional Ethics Committee (Dnr: 2013/2:11) and all participants provided written informed consent prior to the study procedures. The study was conducted according to principles outlined in the Helsinki Declaration.

### Patient population

Adult patients diagnosed with PAH or CTEPH and with symptoms of dyspnoea were considered for the study. Dyspnoea was assessed on a 5-point Likert-type scale; if the Likert-type score was two or higher at rest or at light exercise, the patient was considered eligible for the study. Additional *inclusion criteria* were: stable on disease-specific treatment for at least 3 months and no planned change in treatment during the coming 6 months, understand instructions on how to use the study equipment and willingness to participate in the study procedures and sign the study consent form. *Exclusion criteria were* symptomatic systemic hypotension and simultaneous inclusion in any clinical intervention trial.

### DGB

**Figure 1.**
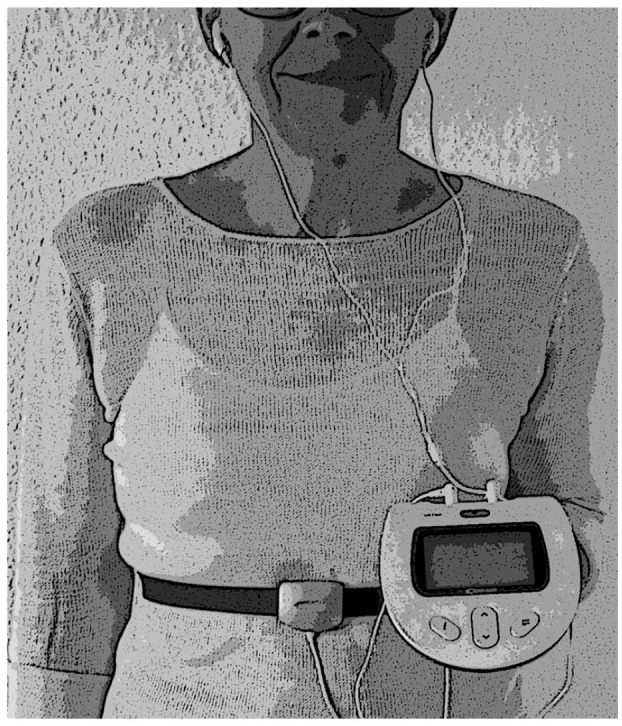
Respiratory rate modulation aimed to slow the respiratory rate by generating musical tones in response to the patient’s own breathing movements (RESPeRATE, InterCure Ltd., Lod, Israel). A belt-type sensor placed around the chest or upper abdomen recorded breathing movements in real time that was fed back to the device. The device generated musical tones for inhalation and exhalation that the patient listened to with headphones. The tones guided the breathing to become slower. Permission to publish the figure was given by RESPeRATE, 2breathe Technologies Ltd.

RRM was accomplished using a device (RESPeRATE, 2breathe Technologies Ltd. RESPeRATE Inc., Newark, NJ, USA) that aimed to slow the respiratory rate by generating musical tones in response to the patient’s own breathing movements. Breathing movements were recorded in real time via a belt-type sensor placed around the chest or upper abdomen. The data were processed and used for feedback as well as stored in a battery-operated, computerized device. The same device also generated the musical tones that the patient listened to with headphones (Figure 1).

During an initial phase of approximately 1 min, the patient’s spontaneous breathing pattern was identified. Based on this information, the RRM phase was initiated using different musical tones for inhalation and exhalation. The goal was to progressively slow the respiration rate to <10 breaths/minute. This was accomplished in an individualized way by continuous feedback to the device about the patient’s own respiratory rate during the session. In addition to slowing the respiratory rate, the musical tones increased the exhalation time relative to the inhalation time^12^ mimicking the natural breathing pattern. The device was programmed to shut off automatically after 20 min. All patients received detailed instructions on how to apply and use the RRM-device and the first experience using the device was supervised by research staff at the hospital. Patients were asked to use the device for 20 min, in a sitting or supine position, once a day for 3 months.

### Measurement of respiratory rate and breathing patterns

A MediByte device (Braebon Medical Corporation, Ogdensburg, NY, USA) was used to record respiratory rate. This was accomplished by two elastic belts (strain gauge), one positioned around the upper thoracic body, just below the armpits and one positioned around the belly, just below the rib cage. Body position was measured by a sensor positioned inside the MediByte case. The measured data were recorded and stored continuously in the MediByte device and downloaded to a computer with a device-specific analysing software at the hospital. After applying the elastic belts, the patients were asked to perform standardized breathing exercises that affected the respiratory rate. Control exercises were: slow and rapid breathing guided by metronome for 2 min each followed by reading a text aloud for 2 min. MediByte data were then recorded during the 6-min walk tests,^13^ RRM at all visits and ambulatory for 24 h after the baseline and intervention visits. During the visit, the respiratory rate was calculated at rest (first minute of RRM), last minute of 6-min walk test, 2 min after stopped walking and in the ambulatory setting at midnight, at 2 a.m., 4 a.m. and 1 h before awakening. During the recording, patients kept a diary where they noted the time of major activities during the day, going to bed, bathroom visits at night and time of awakening as well as arising in the morning.

### Clinical measurements performed at visits

Clinical measurements included systemic blood pressure, weight, biochemistry, dyspnoea score (Likert-type 1–5 scale^14^), QoL measures and 6-min walk distance including measurements of dyspnoea (Borg scale 1–10^15^) and fatigue (Borg scale 6–20^16^) before and after the walk. In addition, patients were asked to answer yes or no to three non-validated questions: (1) if the device had been helpful in controlling their symptom of dyspnoea, (2) would they like to continue to use the device or have access to it in the future and (3) would they recommend other patients to try the device.

*The 6-min walk test* is a standardized and a well-evaluated tool to measure exercise capacity.^13^ It is considered a submaximal exercise test and is part of the routine evaluation in patients with PAH and CTEPH.^1^ The walking speed is decided by the patient, but the goal of the test is to walk as long distance as possible during 6 min.

*The EuroQol-5 dimension (EQ-5D*;^17,18^ *non-commercial licence*) includes five questions that represent five health dimensions: mobility, self-care, usual activities, pain/discomfort and anxiety/depression. Each dimension can be rated from 1 to 3 and will give a measure of an individual’s health-related quality of life (HRQoL). EQ-5D is converted into a single summary index, where the best possible health is 1.00.^19,20^ The EQ-5D also includes a self-rated health status scale (EuroQol Visual Analogue Scale (*EQ-VAS*)) consisting of a 20-cm vertical scale anchored with 100 at the top (best imaginable health state) and with 0 at the bottom (worst imaginable health state). Patients are asked to mark their current state of health on the vertical line in relation to perfect and worst health.

*The Cambridge Pulmonary Hypertension Outcome Review* (*CAMPHOR;*^21,22^ *non-commercial licence*) measures the QoL (25 questions) in parallel with symptoms (25 questions) as well as levels of physical activity (15 questions). Symptom and QoL items are both scored as ‘yes/true’ = 1 and ‘no/not true’ = 0, possible maximal score is 25 for each item. Physical activity has three response levels (score 0–2), giving a possible maximal score of 30. The sum for each dimension as well as a total sum is used in the evaluation. A high sum indicates a worse QoL.

## Statistical methods

The Student’s *t*-test was used for statistical comparisons of continuous variables between the study groups. All analyses were carried out by use of the SAS statistical software (SAS 9.4). A *p*-value of 5% or less was considered as significant.

The study was constructed as a pilot study without a power calculation as no prior data on the effect of RRM using DGB was available in this patient population when the study was planned or executed.

## Results

Eighteen patients were included in the study and of those 14 (PAH = 9 and CTEPH = 5) completed the baseline and intervention visits. Thirteen of these patients also completed the end visit. One patient could not do the walk test at all and another three patients did not do the walk test at the end visit.

Mean age was 71 ± 14 years, 11 were women and the mean time from diagnosis was 4.0 ± 2.7 years. All patients were treated with disease-specific drugs, seven with single- and seven with combination treatment (Table 1). Two patients were on home oxygen therapy. No patient was treated with a calcium channel blocker. There were no changes in disease-specific medical treatment during the course of the study. In one patient, ambulatory recording of respiratory rate revealed respiratory pauses and the patient was referred for further investigation at a sleep laboratory.

**Table 1.** Patient characteristics.

Characteristics (*n* = 14)	
Age (years)	71 ± 14
Female sex	11 (64)
Diagnosis	
IPAH	8 (57)
APAH-CTD	1 (7)
CTEPH	5 (36)
Smoker	
Never	4 (29)
Current	1 (7)
Previous	9 (64)
PAH-specific treatment	
Endothelin receptor antagonist	4 (29)
Phosphodiesterase 5 inhibitor	3 (21)
Endothelin receptor antagonist + phosphodiesterase 5 inhibitor	6 (43)
Prostacyclin analogue* + endothelin receptor antagonist	1 (7)

IPAH: idiopathic pulmonary arterial hypertension; APAH-CTD: pulmonary arterial hypertension associated with connective tissue disease; CTEPH: chronic thromboembolic pulmonary hypertension; PAH: pulmonary arterial hypertension; SD: standard deviation.

Data are shown as mean ± SD or number (%).

* Implantable drug pump.

After 3 months using the DGB, the World Health Organization (WHO) functional class and dyspnoea score had improved while the respiratory rate at rest and N-terminal pro b-type natriuretic peptide (NT-proBNP) had decreased (Table 2). QoL by EQ-VAS improved, however, not significant (Table 2). There was no change in 6-min walked distance; however, fatigue after the walk as well as the respiratory rate at the end of the test and at 2 min after the 6-min walk decreased (Table 2).

**Table 2.** Measurements at baseline and after 3 months with respiratory modulation (intervention).

	Baseline (*n* = 14)	Intervention (*n* = 14)	*p*-value
WHO functional class	2.4 ± 0.5	2.1 ± 0.3	*0.040*
Weight (kg)	68 ± 16	68 ± 17	*0.218*
Systolic blood pressure (mm Hg)	128 ± 27	127 ± 21	*0.764*
Diastolic blood pressure (mm Hg)	73 ± 12	72 ± 14	*0.616*
Heart rate (bpm)	72 ± 8	66 ± 9	*0.081*
Respiratory rate at rest (br/min)	12 ± 4	9 ± 2	*0.013*
Dyspnoea score	3.1 ± 1.1	2.4 ± 0.8	*0.014*
NT-proBNP	745 ± 698	582 ± 551	*0.043*
IL-6	3.0 ± 1.3	2.5 ± 0.8	*0.344*
Ambulatory respiratory rate			
At midnight (br/min)	16 ± 5	18 ± 4	*0.088*
At 2 a.m. (br/min)	16 ± 6	17 ± 3	*0.575*
At 4 a.m. (br/min)	17 ± 6	17 ± 2	*0.886*
One h before awakening (br/min)	15 ± 5	17 ± 2	*0.467*
Six-min walk test			
Walked distance (m)	413 ± 89	413 ± 105	*0.991*
Dyspnoea after (Borg scale)	5.0 ± 1.5	4.8 ± 2.0	*0.513*
Fatigue after (Borg scale)	3.7 ± 2.3	2.6 ± 1.8	*0.036*
Respiratory rate at end of walk (br/min)	38 ± 10	36 ± 8	*0.367*
Respiratory rate 2’ after walk (br/min)	23 ± 5	19 ± 4	*0.002*
Quality of life			
CAMPHOR (sum score)	22 ± 9	22 ± 11	*0.966*
Symptom (score)	8 ± 4	8 ± 4	*0.522*
Physical activity (score)	7 ± 3	8 ± 4	*0.517*
Quality of life (score)	6 ± 5	6 ± 5	*0.924*
EQ-5D index	0.79 ± 0.21	0.80 ± 0.11	*0.832*
VAS (score)	65 ± 14	71 ± 18	*0.131*

WHO: World Health Organization; NT-proBNP: N-terminal pro b-type natriuretic peptide; IL-6: interleukin 6; CAMPHOR: Cambridge Pulmonary Hypertension Outcome Review; EQ-5D: EuroQol-5 dimension; VAS: Visual Analogue Scale; SD: standard deviation.

Data are shown as mean ± SD or number (%).

The answers to the three general questions about the usability of the DGB were positive with a majority (*n* = 12) saying yes to the device being helpful in controlling their symptom of dyspnoea, that they would they like to continue to use the device or have access to it in the future and that they would recommend other patients to try the device.

**Figure 2.**
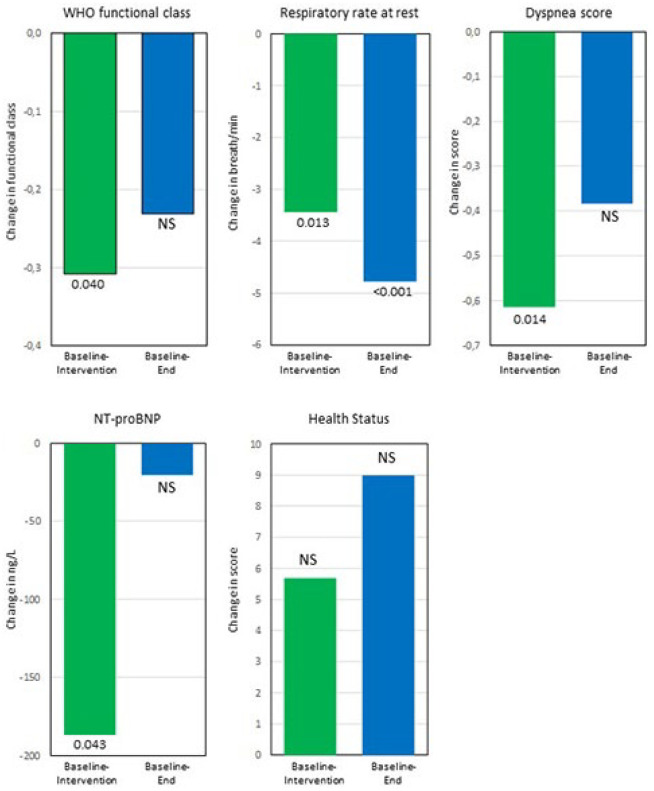
The mean change from baseline to intervention and from baseline to end for those variables that showed a statistical significant change from baseline to intervention. All variables improved or remained unchanged at the end visit compared to baseline. At the end visit, World Health Organization (WHO) functional class, health status by EQ-VAS and dyspnoea score was available from 13 patients, NT-proBNP from 12 patients and respiratory rate at rest from 9 patients. Baseline: start of study; intervention: after 3 months using RRM; end: 3 months without RRM after completing intervention.

**Figure 3.**
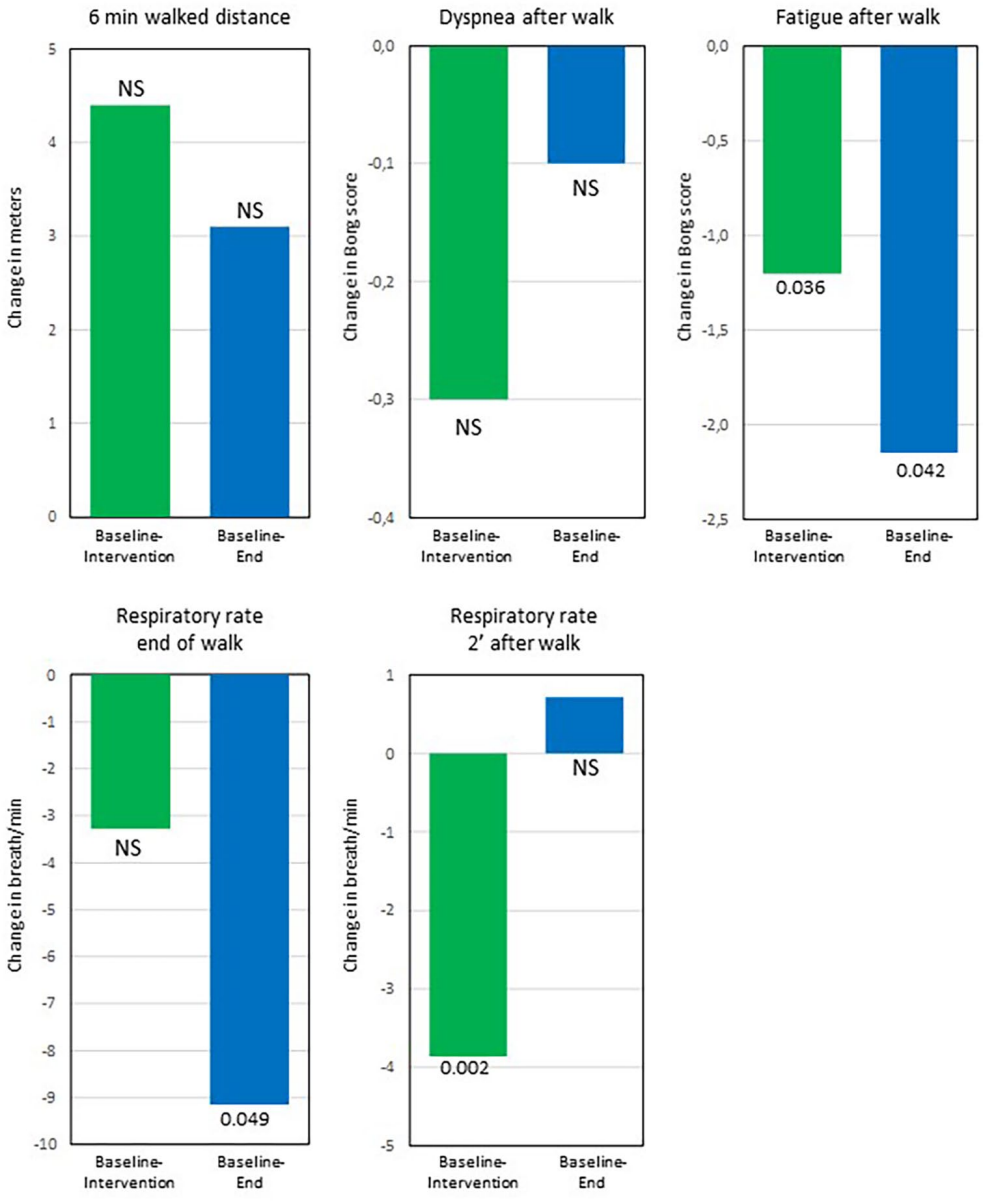
The mean change from baseline to intervention and from baseline to end for variables related to the 6-min walk test (*n* = 10). Despite the walked distance not increasing significantly, the respiratory rate at end of walk test as well as fatigue and respiratory rate after the walk improved significantly from baseline to intervention and remained improved or unchanged at the end visit. Baseline: start of study; intervention: after 3 months using RRM; end: 3 months without RRM after completing intervention.

At the end visit, 3 months after stopping DGB, the respiratory rate at rest as well as fatigue and respiratory rate after the 6-min walk test remained decreased compared to baseline (Figures 2 and 3).

## Discussion

Patients that applied DGB for 3 months improved symptoms of dyspnoea and lowered the respiratory rate at rest as well as after exercise, suggesting a respiratory modulation effect. There was no adverse response to RRM by DGB in any of the measured variables. After 3 months without DGB, all variables remained unchanged or improved compared to baseline, but only changes in the respiratory rate at rest as well as respiratory rate and fatigue after exercise remained significant.

Dyspnoea is a complex symptom and even in the same patient, there might be several reasons for this symptom.^4^ It might be a subjective experience of breathing discomfort for the patient, where the breathing is not matched by adequate pulmonary ventilation.^23^ This discomfort can vary in its intensity. The American Thoracic Society (ATS) has described dyspnoea as ‘an experience derived from interactions among multiple physiological, psychological, social, and environmental factors. It may also induce secondary physiological and behavioural responses’.^23^ Thus, dyspnoea should be considered as a multifaceted phenomenon that includes physical factors as well as secondary elements caused directly by the distress associated with the difficulty in breathing.

The four components of breathing: ventilation, respiratory regulation, exchange and transport of gases can all lead to a feeling of breathlessness or dyspnoea.^4^ It also differs from other vital functions by both having automaticity activated by centres located in the brainstem as well as voluntary regulation by signals initiated in the cortex. In other words, we cannot decide to stop breathing, but we can decide to alter our breathing rate and patterns. RRM by DGB aims to mimic the natural breathing pattern by consciously slowing the respiratory rate and increase exhalation time relative to inhalation time.^12^ In this study, RRM was accomplished with a device that, in a few minutes, guided the patients to breathe slower and deeper.^9–12^

The mechanism behind the improvement in symptoms and decreased respiratory rate seen in this study cannot be explained by the undertaken measures. This was not part of the study’s aim nor its design. Nevertheless, there are several plausible explanations that may have contributed to the improvement, such as a positive baroreflex sensitivity response,^6,9^ an improved thoracic muscle strength,^24^ and/or a better coordination between the diaphragm and inhalation/exhalation movements.^12^ The decrease in NT-proBNP, heart rate and respiratory rate seen after the intervention might indicate a positive baroreflex sensitivity response, but studies including mechanistic measures are needed to explore this further.

This study showed that after using DGB for 3 months, a vast majority of the patients reported an improved ability to control their feeling of dyspnoea compared to the time prior to the study. This is likely the most clinically significant result of the study and might have contributed to the improvement in WHO functional class and health status measured by the EQ-VAS; however, the latter was not significant. For patients with PAH or CTEPH, even a low level of activity might cause dyspnoea and fatigue as they quickly might reach their limit in cardiopulmonary reserve. Thus, as patients take control of the feeling of dyspnoea, this might have a significant effect on how they can perform their daily tasks.

A low respiratory rate has been suggested to be physiologically beneficial.^6^ After using DGB for 3 months in this study, the respiratory rate was lowered at rest as well as at midnight; however, the latter was not significant. Respiratory rate was also lower after the walk test, despite walking the same distance during the test. This finding supports the patients’ experience of better controlling their dyspnoea.

This study, in conjunction with the results previously presented by Matura et al.,^11^ indicates that RRM using DGB might have a beneficial effect on symptoms of dyspnoea in patients with PAH or CTEPH. Both studies have small study populations and show only discreet positive effects on symptoms and inflammatory markers, but taken together, they support the idea that RRM by DGB might benefit patients with PAH and CTEPH. While the results from these two studies should be interpreted with caution, they do support the notion that improved control of dyspnoea might give patients a better understanding of the symptom and possibly even prevent dyspnoea from occurring. This should encourage larger, controlled multicentre studies with a design aimed at not only investigating if the result, decreased dyspnoea, can be repeated on a larger scale but also investigating possible underlying mechanisms.

## Limitations

The study was a single-centre study designed to investigate if RRM could improve the symptom of dyspnoea by DGB. No measures, such as right heart catheterization or echocardiography, that investigate the underlying cause of dyspnoea or any physiological mechanisms that might have been altered by RRM were performed in the study. In addition, analyses of the effect on the 6-min walk test would have been more complete if it had included measures of SaO_2_ and heart rate.

The study should be regarded as a pilot study. At the time of planning and conducting the study, no prior data on RRM using DGB were available for this patient population, and thus, no power calculation was performed to justify the sample size. For the same reason, no control group was included. With two studies, Matura et al.^11^ and the present, showing positive trends in utilizing RRM to alter dyspnoea, future studies can be planned as a case–control intervention study and with a sample size calculation.

The placebo effect of being included in a study should be taken into account when interpreting the results. However, all patients were seen at the PAH/CTEPH-specialist clinic where they had regular contact and access to the specialist nurses. This might lessen the placebo effect of being included in a study. In addition, the decrease in NT-proBNP and IL-6 (not significant) as well as respiratory rate indicates that there might be a non-placebo effect.

The compliance of using the DGB cannot be reported upon due to technical problems with this function in the device. This function was altered by the manufacture to cover the whole study period of 3 months; however, no data at all were stored. That this affected all devices were not discovered until several patients had undergone RRM with DGB. As correcting this would have meant returning all devices back overseas to the manufacturer, the study was continued without any hard measure of compliance. The patients self-reported compliance in using the DGB, but this could not be verified.

## Conclusion

Patients with PAH or CTEPH that used DGB for 3 months improved symptoms of dyspnoea and lowered the respiratory rate at rest and after exercise. This could be of importance as even a modest decrease in symptoms and a small increase in physical capacity might make an important change in the QoL for patients with PAH or CTEPH.
